# Influence of the anaesthetist´s perception on the evaluation of recovery quality in horses undergoing general anaesthesia

**DOI:** 10.1016/j.vas.2026.100899

**Published:** 2026-10-15

**Authors:** Leire Benavente-Sánchez, Marta Villalba-Díez, Carla Jaen-Garcia, Isabel Santiago-Llorente, María Villalba-Orero

**Affiliations:** a Department of Animal Medicine and Surgery, Faculty of Veterinary Medicine, Universidad Complutense de Madrid, Spain; b Complutense Veterinary Teaching Hospital, Universidad Complutense de Madrid, Spain

**Keywords:** Horses, Anaesthesia, Recovery, Perception

## Abstract

**Background:**

In horses, anaesthetic recovery is evaluated using subjective scales. Previous studies examined the influence of scale type and evaluator-related factors but the impact of operator perception (unconscious bias) has received limited attention.

**Objectives:**

To analyse the influence of operator perception on recovery scoring.

**Methods:**

Anaesthetic recoveries at an equine hospital (May 2019-May 2023) were assessed in real time (RT) by attending anaesthetists and retrospectively using video recordings (VR) by a single evaluator. Horses aged 1 – 21 years undergoing a single anesthetic procedure with complete video recordings were included. Recovery quality was scored using a simple scale (SS; 1 = excellent to 5 = dangerous) and a detailed scale (DS; 0 = very violent to 5 = no ataxia). RT and VR were compared using the Wilcoxon signed-rank test. A secondary analysis included only cases evaluated by the same anaesthetist in both conditions. Exploratory analyses assessed influences of time of day, procedure, anaesthesia duration, number of standing attempts and grouped recovery categories. Analyses were performed using SPSS (IBM) (p < 0.05).

**Results:**

Of 262 recoveries, 154 met inclusion criteria. VR scores were significantly worse than RT assessment for both scales [SS: 2 (IQR: 1); DS: 4 (IQR: 1)], including same-evaluator cases [SS: 2 (IQR: 2); DS: 4 (IQR: 1)]. Discrepancies persisted across most subgroups but were not observed in unsatisfactory recoveries, or for DS scores during nighttime or horses presenting ≥3 standing attempts.

**Conclusions:**

Anaesthetists tend to assign more favourable scores when evaluating their own patients in RT, suggesting an optimism bias.

## Highlights

• A realistic assessment of the recovery quality in horses is fundamental for evaluating this period.
• Subjectivity associated with the evaluator is also susceptible to unconscious bias (operator perception).
• Real-time recovery assessments exhibit a systematic deviation, tending to be optimistic.

## Introduction

The recovery phase is a critical period in equine anaesthesia, as it carries a high risk of catastrophic incidents (Johnston et al., 2002; Laurenza et al., 2020). Indeed, 25–50% of anaesthesia-related fatalities occur during the recovery period (Dugdale & Taylor, 2016; Young & Taylor, 1993). As recovery is a marked risky period in equine anaesthesia at present, many efforts have been made aiming to improve this anaesthetic phase (Gozalo-Marcilla et al., 2021). A realistic assessment of the recovery quality is fundamental for evaluating this period; therefore, the use of an appropriate method to analyse recovery quality in equids is essential.

Numerous scales have been described to assess the recovery from general anaesthesia in horses. The most used include visual analogue scales (VAS) (Hubbell, 1999), simple descriptive scales (SDS) (Young & Taylor, 1993) and more detailed composite grading scales (CGS) (Donaldson et al., 2000). Although the use of structured or validated scoring systems may improve the consistency of recovery assessment, a degree of subjectivity remains inherent to the evaluation process. Subjectivity hampers repeatability and reproducibility, and must be carefully analysed and taken into account when interpreting the results of scores obtained from the scales. Many studies address these features and how both are influenced by the specific scale used or by specific factors related to the evaluator as experience, fatigue, gender and background^,^(Clark-Price et al., 2017; Farmer et al., 2014; Scarabelli & Rioja, 2017; Suthers et al., 2011; Vettorato et al., 2010). Nevertheless, subjectivity associated with the evaluator is also susceptible to unconscious bias (operator perception) (Pollock, 2020); but studies seldom analysed this subconscious bias in experienced observers or the factors that may influence this operator perception.

As the replicability of findings is an essential prerequisite in clinical research (Leichsenring et al., 2017), understanding unconscious bias in the assessment of the recovery phase of clinical anaesthesia in horses is paramount to reducing subjectivity in the recovery scales used. In this study, we aimed to analyse the impact of changes in operator perception on the anaesthesia recovery assessment outcome, by comparing real-time evaluations conducted by the attending anaesthetists during clinical management with retrospective assessments based on video recordings conducted by a separate evaluator. In addition, we aimed to determine external factors that may influence operator perception.

We hypothesized that recovery scores would systematically differ between real-time and retrospective video assessments, reflecting the influence of contextual factors in operator´s perception.

## Material and methods

### Animals

This study includes the anaesthetic recoveries of all horses admitted to the Complutense Teaching Equine Hospital and anaesthetized between May 2019 and May 2023, whose recoveries were evaluated immediately after recovery and for which a complete video recording of the recovery was preserved for retrospective analysis.

This retrospective study did not require any deviation from routine medical practice and all owners signed an informed consent which included permission to use their animal’s clinical data for research purposes.

### Study design

All anaesthetic records were reviewed to obtain the real-time recovery scores assigned by one of the six anaesthetists responsible for the routine clinical anaesthetic management of the hospital. Systematically, two recovery scoring systems were used. One was a simple scale (SS) with scores using the following features: 1- excellent; 2- good; 3- regular; 4- poor and 5- danger (Wagner et al., 2008). The other scale used was more detailed (DS; detailed scale (Young & Taylor, 1993), with the following scores: 0- very violent (may die), present damage and prolonged struggle to achieve standing position, unable to stand; 1- excitement when recumbent, many unsuccessful attempts to stand, severe ataxia and lying down after standing, walking in circles, high risk of damage; 2- excited and cycling when recumbent, many attempts to achieve standing position, ataxia while standing, may fall, can be dangerous and may present injury; 3- slight ataxia and faltering; several attempts to stand, ataxia while standing; 4- slight ataxia and faltering, one or two attempts to stand, no instability in standing position; 5- no ataxia, standing position achieved on the first attempt without difficulty.

Additionally, video recordings of the anaesthetic recovery phase were extracted using VideoPad® and stored by an investigator who was not involved in their retrospective assessment. The recordings were subsequently evaluated by a single experienced anaesthetist, who applied the same two assessment scales described above. During retrospective video evaluation, the assessor did not have access to procedural variables (type of procedure, duration of anaesthesia, or time of day) and was unaware of which anaesthetist had performed the case.

All horses were positioned in right lateral recumbency and allowed to recover freely in a padded recovery stall, with the lights on and without eye covering. Horses received and α2-adrenoreceptor agonist for sedation (romifidine at 0.02 mg/kg). Supplemental oxygen was initially administered using a demand valve, while subsequent nasal oxygen insufflation was provided when considered necessary by the attending anaesthetist.

All the recoveries were recorded using a fixed video camera positioned in the same corner of the recovery box, providing a top and lateral view of the horse throughout the recovery period. Video recordings had a resolution of 1280×780 pixels at 12 frames/s and did not include audio. Real-time observations were performed by the attending anaesthetists from an observation window located in a different corner of the recovery area, but they also had access to real time recording of the video camera in a screen next to the recovery box. Both perspectives allowed continuous observation of the entire recovery process.

Recoveries were excluded if: horses were younger than 1 yo or older than 21 yo; horses underwent multiple anaesthetic procedures (only the first was included); or if the video recordings were damaged or incomplete, hindering the visualization of all recovery stages.

### Data registered

In addition to the two recovery scores, demographic and clinical data were also collected: age (yo), sex (male, female), breed, body weight (kg), time of day (hour at which the anaesthesia recovery evaluation was performed: daytime (8 am to 9 pm), nighttime (9 pm to 8 am), ASA (American Society of Anesthesiologists) grade, type of procedure (elective or emergency), anaesthesia duration (minutes, from induction until standing) and the number of attempts to stand.

### Statistical analysis

Statistical analyses were performed using SPSS (IBM; Version 29.0.2.0), with statistical significance set at p < 0.05. Normal distribution was assessed using the Kolmogorov-Smirnov test. Parameters following a normal distribution were presented as mean ± standard deviation (SD), and those that did not follow normal distribution were presented as median and interquartile range (IQR).

Given the ordinal nature of the scales and the non-normal distribution of the recovery scores, the nonparametric Wilcoxon signed-rank test was used to compare the scores obtained during the real-time (RT) assessments with those obtained retrospectively from the video recordings (VR). To assess the effect of evaluation context (RT *vs* VR) a primary analysis included RT evaluations performed by multiple anaesthetists and VR evaluations conducted by a single observer (n = 154) Additionally, to explore whether inter-observer variability influenced the results, a second comparison was conducted using only recovery scores assigned by the same evaluator for both RT and VR assessments (n = 121). In order to highlight inconsistencies, graphs were generated to illustrate negative and positive differences, as well as ties, in the scores given, using VR as reference.

To explore factors potentially influencing discrepancies between RT and VR recovery scores, additional exploratory analyses, including all recoveries, were performed using the Wilcoxon signed rank test after grouping the data according to selected clinical parameters. These included time of day (daytime: 8am – 9pm; nighttime: 9pm – 8am), type of procedure (elective or emergency), anaesthesia duration (short: ≤120 min or long: <120 min), number of attempts to stand (1–2 or ≥3) and overall recovery quality classifying scores as satisfactory = scores 1 – 2 for the SS and 4 – 5 for the DS; regular = score 3 for both scales; or unsatisfactory = scores 4 – 5 for the SS and 0 – 2 for the DS.

Effect sizes for the Wilcoxon signed-rank tests were calculated using the formula r=Z/√N. To provide an approximate estimate of statistical power, the nonparametric results were additionally approximated within a parametric framework by converting the effect size to Cohen’s *d* for a paired *t-*test.

## Results

### Descriptive analysis

During the study period, a total of 262 anaesthetic recovery procedures were performed, and corresponding video recordings were obtained. The following cases were removed based on the exclusion criteria: 8 cases due to age being <1 yo; 16 recoveries corresponding to a second anaesthesia for the same individual; 41 due to video damage that impaired visualization; 41 cases with incomplete video recordings where not all the recovery period was fully visible; and 2 cases lacked a recovery evaluation score. After these exclusions, 154 recoveries were included in the present study, with the RT evaluations performed by six different anaesthetists. One of these anaesthetists conducted 121 RT evaluations and also performed all the retrospective VR evaluations.

Of the 154 procedures, 133 (86.3%) anaesthesia occurred during the day and 21 (13.7%) during the night; 117 (76.0%) were elective procedures whereas 37 (24.0%) were emergency procedures (all acute abdominal syndrome). The median duration of anaesthesia was 121 (IQR: 50) minutes, and horses made 1.9 ± 1.2 attempts to stand.

The median recovery score recorded during RT evaluation was 2 (IQR: 2) for the SS and 4 (IQR: 1) for the DS. Among these horses, 100 (65.0%) horses showed satisfactory recoveries, 39 (25.3%) showed regular recoveries and 15 (9.7%) showed unsatisfactory recoveries. The median recovery score, when the evaluation was retrospectively performed using VR, was 2 (IQR: 2) for the SS and 4 (IQR: 1) for the DS.

### Reproducibility of real-time *vs* retrospective recovery assessment

Recovery scores assigned during RT evaluation were statistically different for both scales compared to those assigned retrospectively after the VR review (p < 0.0001 for SS; p < 0.0001 for DS). More specifically, poorer recoveries were recorded when the assessment was performed retrospectively using the recorded videos (Fig. 1A and 1B). When using the SS and comparing it with the RT assessment, 62 horses received lower scores (indicating worse recovery) upon retrospective video review, while only 14 received higher scores. Using the DS, reliability did not improve, and a similar result was observed. In the retrospective review (VR), 63 horses received higher scores (indicating worse recoveries) compared to the RT evaluations.

**Fig. 1:**
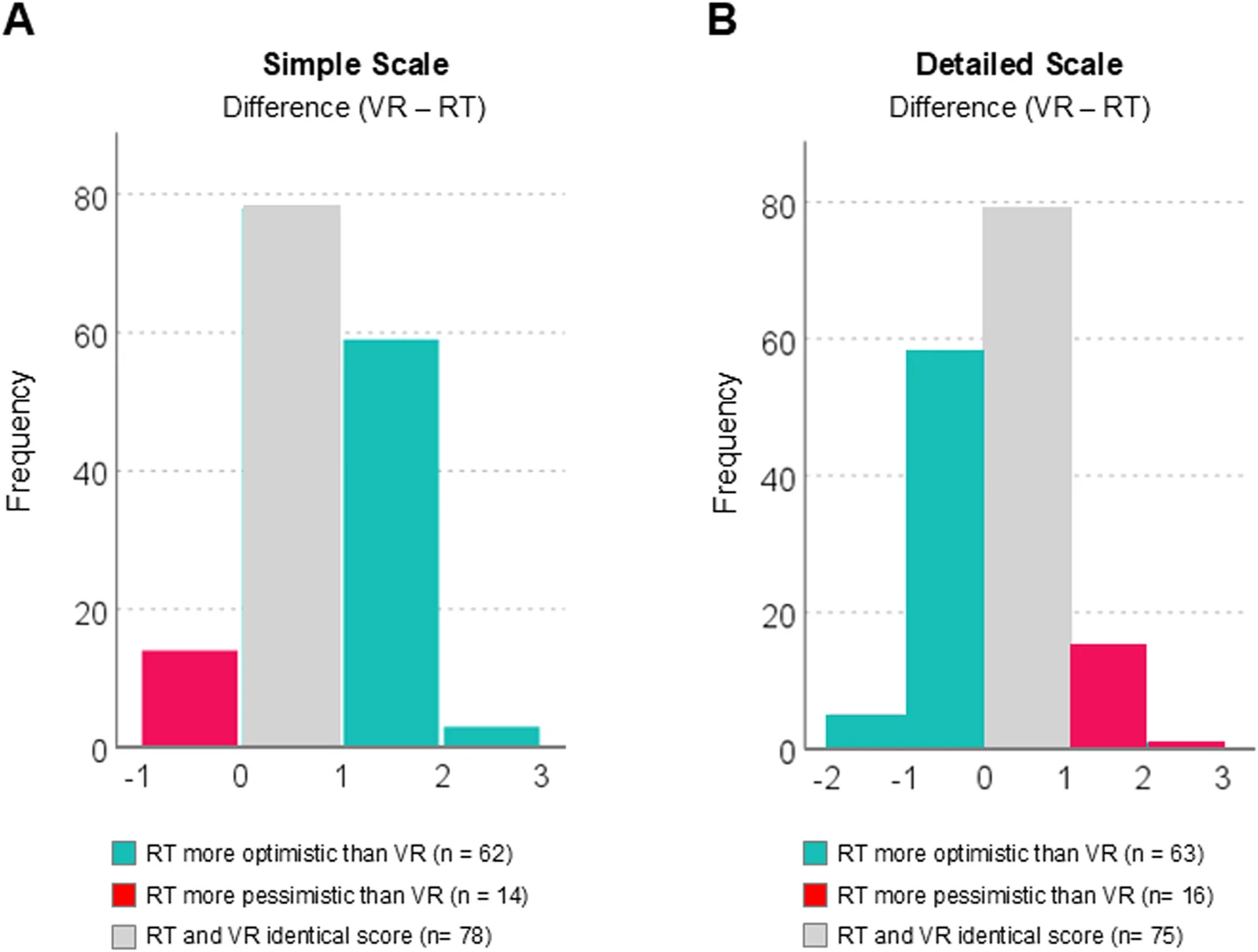
Histogram showing the distribution of score differences between video record review (VR) and real – time recovery assessment (RT). Differences were calculated as VR-RT and analysed using the Wilcoxon signed-rank test. Bars in green indicate cases where RT assessment was more optimistic than the video review, whereas red bars indicate cases where the RT assessment is more pessimistic. Grey bars represent identical scores. For the **simple scale (A),** lower scores indicate better recovery quality. For the **detailed scale (B),** higher scores indicate better recovery quality. Significant differences were found between RT and VR scores for both scales (SS: p < 0.0001; DS: p < 0.0001).

To account for potential clustering due to multiple anaesthetists performing the RT evaluations, an additional comparison was conducted using only one evaluator for both, RT and VR evaluations (n = 121). In this subset, scores obtained during the retrospective VR evaluation were also worse than those recorded during RT for both scales (Fig. 2A and 2B; p < 0.001 for SS; p < 0.001 for DS).

**Fig. 2:**
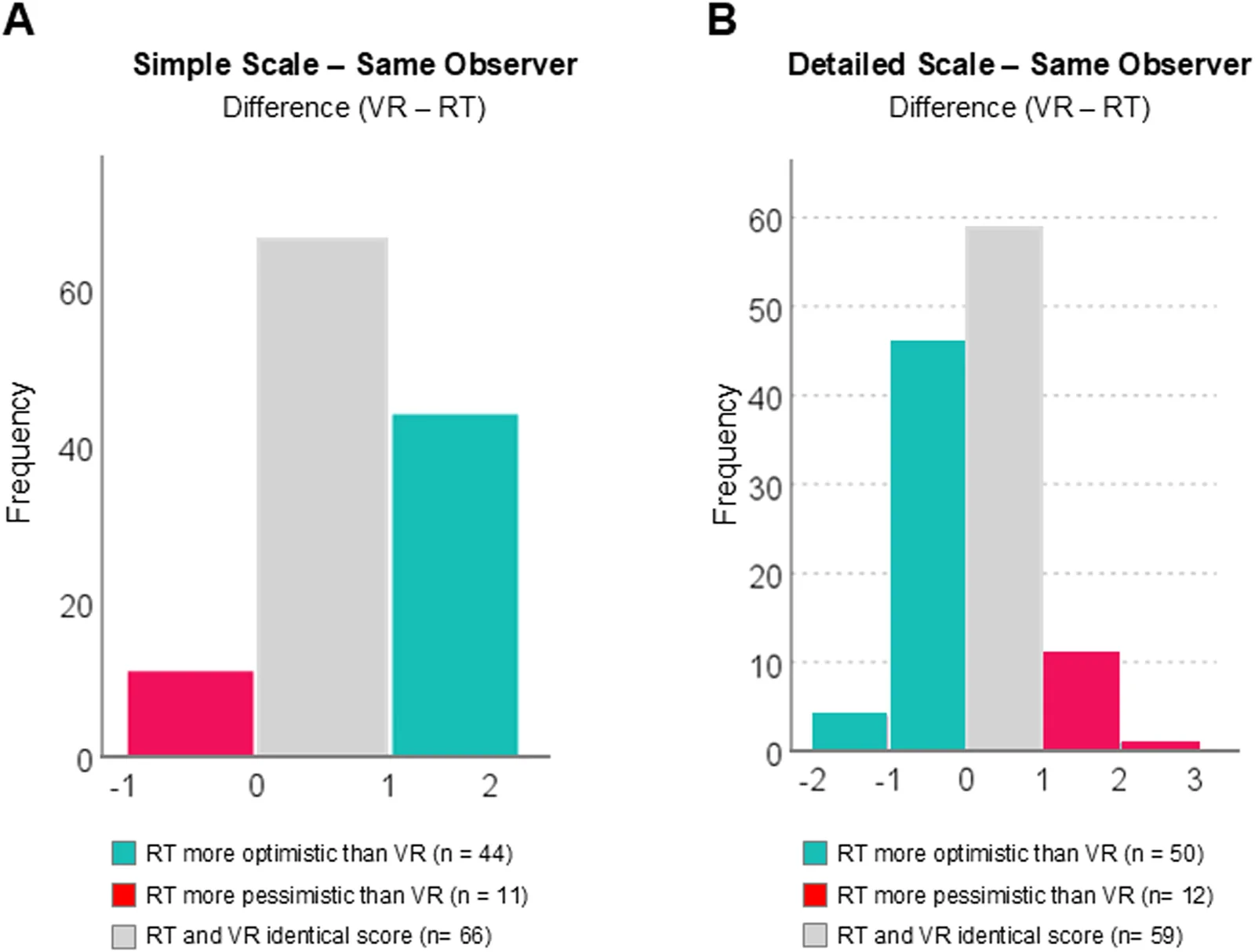
Histogram showing the distribution of score differences between video record review (VR) and real-time recovery assessment (RT) for cases in which both assessments were performed by the same anaesthetist. Differences were calculated as VR-RT and analysed using the Wilcoxon signed-rank test. Bars in green indicate cases where RT assessment was more optimistic than the video review, whereas red bars indicate cases where the RT assessment is more pessimistic. Grey bars represent identical scores. For the **simple scale (A),** lower scores indicate better recovery quality. For the **detailed scale (B),** higher scores indicate better recovery quality. Significant differences were found between RT and VR scores for both scales (p < 0.001 for SS; p < 0.001 for DS).

### Factors influencing reproducibility

Additionally, to assess factors that may contribute to the lack of reproducibility previously observed between RT scores and those obtained retrospectively, using VR, we analysed differences by grouping the recoveries by time of the day, type of procedure, anaesthesia duration, number of attempts to stand and recovery quality (Table 1).

**Table 1:** Comparison of Real-Time and Retrospective (based in video recordings) recovery scores grouped by various Factors in horses undergoing general anaesthesia (N = 154).

Recovery scores								
**Factors**	**Simple scale**				**Detailed scale**			
		***Real-Time***	**Video Recording**			***Real-Time***	**Video Recording**	
	***n***	Median (25th/75th)	Median (25th/75th)	***p***	***n***	Median (25th/75th)	Median (25th/75th)	***p***
Time of the day								
Daily	*133*	2 (1/3)	2 (2/3)	**<0.0001**	*133*	4 (3/4)	4 (3/4)	**<0.0001**
Nightly	*21*	3 (2/3)	3 (2/4)	**<0.05**	*21*	3 (3/4)	3 (2/4)	n.s.
Procedure								
Elective	*117*	2 (1/3)	2 (2/3)	**<0.0001**	*117*	4 (3/4.5)	4 (3/4)	**<0.0001**
Emergency	*37*	2 (1.5/3)	3 (2/4)	**0.001**	*37*	3 (3/4)	3 (2/4)	**<0.05**
Anesthesia duration								
≤120 min	*75*	2 (1/3)	2 (2/3)	**<0.0001**	*75*	4 (3/5)	4 (3/4)	**<0.001**
>120 min	*79*	2 (2/3)	2.5 (2/3)	**<0.001**	*79*	4 (3/4)	3 (3/4)	**<0.001**
Attempts to stand								
1–2	*111*	2 (1/2)	2 (2/2)	**<0.0001**	*111*	4 (4/5)	4 (3/4)	**<0.0001**
≥3	*40*	3 (3/4)	4 (3/4)	**<0.05**	*40*	3 (2/3)	2 (2/3)	n.s
Recovery quality								
Satisfactory	*100*	2 (1/2)	2 (2/2)	**<0.0001**	*93*	4 (4/5)	4 (3.25/4)	**<0.001**
Regular	*39*	3 (3/3)	3 (3/4)	0.180	*41*	3 (3/3)	3 (2/3)	**<0.05**
Unsatisfactory	*15*	4 (4/4)	4 (4/4)	0.180	*20*	2 (2/2)	2 (1/2)	n.s

n.s., non-significance.

Real-time recoveries scored during the day received better ratings compared to those given when reviewing the videos afterward using both scales (Fig. S1A and S1B; p < 0.0001 for SS; p < 0.0001 for DS). A similar result was observed at night when the SS was used (Fig. S1C; p < 0.05). However, retrospective scores from VR did not differ significantly from those obtained during RT evaluations when the DS was applied (Fig. S1D).

For both scales, elective procedures received worse scores during the retrospective VR review compared to the RT evaluation (Fig. S2A and S2B; p < 0.0001 for SS; p < 0.0001 for DS). Similarly, horses undergoing anaesthesia for emergency procedures received worse scores during RT evaluation than those assigned when reviewing the VR retrospectively (Fig. S2C and S2D; p = 0.001 for SS; p < 0.05 for DS).

Retrospective video review (VR) also resulted in poorer scores for both scales, in short (≤120 min; Fig. S3A and S3B; p < 0.0001 for SS; p < 0.001 for DS) and long procedures (>120 min; Fig. S3C and S3D; p < 0.0001 for SS; p < 0.001 for DS). For horses with two or fewer attempts to stand, worse scores were recorded retrospectively during the VR envisioning compared to the RT evaluation for both scales (Fig. S4A and S4B; p < 0.0001 for SS; p < 0.0001 for DS). In horses with three or more attempts to stand, a similar pattern was observed when using the SS (Fig. S4C; p < 0.05); however, when the DS was applied, recovery scores were comparable between the RT and the retrospective VR evaluation. Similarly, horses classified as having satisfactory or regular recovery quality, generally received poorer scores during the retrospective VR envisioning than during the RT evaluation (Fig. S5A and S5B; satisfactory recoveries: p < 0.0001 for SS; p < 0.001 for DS; Fig. S5C and S5D; regular recoveries: p = 0.180 for SS, p < 0.05 for DS). In contrast, the evaluation was similar for those horses with unsatisfactory recovery quality (Fig. S5E and F; p = 0.180 for SS, p = 0.197 for DS).

## Discussion

This study highlights that real-time recovery assessments conducted by the anaesthetists performing the procedure exhibit a systematic deviation, tending to be more optimistic than evaluations performed retrospectively.

In research, bias refers to the presence of a systematic error in an analysis, resulting in a deviation from the truth (Gerhard, 2008; Grimes & Schulz, 2002); typically arising from the collection and measurement of data (Althubaiti, 2016). Evaluators are inherently susceptible to bias, which cannot be overlooked (Pollock, 2020). Although the bias observed in our study could theoretically influence results in either direction, enhancing or diminishing perceived recovery quality, we found that scores assigned during the procedure consistently indicated better recovery than those based on video recordings, highlighting a systematic optimistic bias *in situ* evaluations. Even when the same evaluator assessed their own recoveries retrospectively, better recovery scores were assigned during the real-time evaluation. Notable differences exist between real-time evaluations of recovery quality in horses and retrospective assessments based on video recordings. Most significantly, real-time evaluators are influenced by both external and internal factors. However, most of the factors studied did not affect the optimistic bias detected in the real-time assessments. Therefore, the lack of reproducibility shown may be attributable to the fact that anaesthetists were evaluating the recoveries of their own patients, which may influence the operator’s perception and introduce an optimistic bias. The recovery phase is a high-risk period in equine anaesthesia (Dugdale & Taylor, 2016; G. Johnston et al., 2002; Laurenza et al., 2020; Young & Taylor, 1993), and is particularly important to the anaesthetist performing the procedure. This may contribute to consistently improving recovery scores, likely due to the well-documented phenomenon in which observer expectations unintentionally influence outcomes (Holman et al., 2015).

Objective measures typically produce more reliable results, whereas subjective metrics are more susceptible to systematic error and bias (Aranda López King et al., 2014). In horses, the scales used to address the recovery phase of anaesthesia are subjective, inevitably introducing a risk of bias, particularly when the evaluator is personally involved in the procedure (Holman et al., 2015). In the present study, two simple descriptive scales were employed to assess recovery. These scales are user-friendly and widely utilized in equine anaesthesia research (Donaldson et al., 2000; Santiago-Llorente et al., 2021; Wagner et al., 2008). Both are described as providing a reliable approach for investigating equine anesthesia recovery, though they remain inherently subjective (Clark-Price et al., 2008, 2017; Donaldson et al., 2000). In our work, when compared to the SS, the DS demonstrated greater consistency between the result registered during real-time evaluations with those assigned retrospectively, evaluating the video recordings. This suggests that more detailed scales contribute to reducing subjectivity and may therefore be more suitable for assessing equine recovery when data is intended for research purposes. In addition, the use of blinding assessments is a keystone to enhance reliability and reduce evaluator bias (Althubaiti, 2016). In clinical anaesthesia, a blind analysis of the recovery phase is not routinely performed. This issue is particularly relevant, given that most retrospective clinical studies aiming to reduce mortality and morbidity in equine anaesthesia rely on unblinded assessments of recovery (G. Johnston et al., 2002). Based on the evidence presented in this study, implementing blinded evaluations of equine anaesthesia recovery is strongly recommended when data is intended for research purposes. Video-recording the recoveries in horses undergoing general anaesthesia not only allows for subsequent blind evaluation but also enables an assessment under constant conditions hence reducing factors that affect operator subjectivity.

Interestingly, the pervasive optimism bias observed appears to dissipate when evaluations are conducted during the night or when horses exhibit indicators of poor recovery quality, as (i) three or more attempts to stand, (ii) recovery classified as of regular quality and (iii) recovery classified as of unsatisfactory quality. This finding suggests that, in more markedly negative circumstances, the tendency toward overly positive perceptions diminishes, resulting in a more realistic and accurate assessment.

In this study, scores derived from video recordings were utilized as the reference for statistical analysis and graph construction. This approach was chosen because video recordings allow for detailed review of the recovery process, including the ability to revisit specific moments, thereby enabling more accurate evaluations.

A limitation of this work is that the involvement of six anaesthetists in scoring the recoveries may have introduced confounding factors, potentially affecting the collection and interpretation of our data and contributing to reduced reproducibility. To avoid this effect, a specific statistical analysis was performed, comparing scores assigned by the same observer, during real-time evaluation and subsequently when reviewing video recordings blindly. Importantly, this analysis yielded similar results. Conversely, the fact that all retrospective video evaluations were performed by a single observer represents an additional potential source of bias and may limit the generalizability of these findings. In addition, complete blinding to patient identity during retrospective video evaluation could not be fully guaranteed, as the evaluator had been part of the clinical team performing the anaesthesia. Therefore, recognition of individual horses or particularly memorable recoveries cannot be excluded and represents a potential source of bias in retrospective scoring. However, this risk was reduced by the long interval between the original anaesthetic procedures and the retrospective assessment, the non-chronological and mixed presentation of the recordings, and the use of detailed scoring systems requiring assignment of specific scores based on multiple clinical features. Furthermore, the conditions of retrospective video assessment were different from those of real-time observation. The perspective of the video recordings differed from that of the real – time observer, as the fixed camera was positioned in a different location within the recovery area than the observation window used during clinical monitoring. Differences in viewing angle may have introduced an additional unknown source of bias. In addition, the video recordings did not include audio. Auditory cues associated with struggling, attempts to stand, or contact with the recovery – box walls, which were available to the attending anaesthetists, could contribute to a more negative perception of recovery. Therefore, their absence might be expected to favour a more positive retrospective assessment. However, the opposite trend was observed, with the retrospective assessments generally indicating poorer recovery than real-time assessments. Although the independent effect of auditory information cannot be determined from the present study, its absence is unlikely to explain the direction of the observed differences. Finally, this study was conducted at a single centre, therefore, extrapolation of the findings to other settings may be limited.

In conclusion, this study reveals that recovery evaluations performed by anaesthetists involved in the procedure tend to show an optimism bias, resulting to an overestimation of recovery quality. However, in more markedly negative circumstances, this tendency toward overly positive perceptions decreases, resulting in more realistic and accurate evaluations.

## Funding information

MVD: Ministry of Science, Innovation and Universities; Training of University Professors Grant (FPU22/03984). MVO: Public-private agreement UCM-Boehringer Ingelheim_EM-0000,000,620.

## Data integrity statement

Leire Benavente Sanchez and María Villalba Orero had full access to all the data in the study and takes responsibility for the integrity of the data and the accuracy of the data analysis.

## Ethical animal research

The director of the centre signed an authorisation for research use of Complutense Veterinary Teaching Hospital clinical data.

## Informed consent

Explicit owner consent for inclusion of animals in this study was not obtained. Owners or their agents were made aware that case information may be used for research in general.

## CRediT authorship contribution statement

**Leire Benavente-Sánchez:** Writing – original draft, Investigation, Formal analysis. **Marta Villalba-Díez:** Writing – review & editing, Formal analysis, Data curation. **Carla Jaen-Garcia:** Methodology, Data curation, Conceptualization. **Isabel Santiago-Llorente:** Writing – review & editing, Supervision, Methodology, Investigation, Data curation, Conceptualization. **María Villalba-Orero:** Writing – review & editing, Validation, Methodology, Investigation, Formal analysis, Conceptualization.

## Declaration of competing interest

The authors declare the following financial interests/personal relationships which may be considered as potential competing interests: Maria Villalba-Orero reports financial support and article publishing charges were provided by Complutense University of Madrid. Marta Villalba Orero reports financial support was provided by Spain Ministry of Science Innovation and Universities. If there are other authors, they declare that they have no known competing financial interests or personal relationships that could have appeared to influence the work reported in this paper.

## References

[bib0001] Althubaiti A. (2016). Information bias in health research: Definition, pitfalls, and adjustment methods. Journal of Multidisciplinary Healthcare.

[bib0002] Aranda López King A., Juristo Juzgado N., Dieste Tubio O. (2014). EASE ’14: Proceedings of the 18th International Conference on Evaluation and Assessment in Software Engineering | 18th International Conference on Evaluation and Assessment in Software Engineering (EASE 2014) | 13-14 May 2014 | Londres, Reino Unido, EASE ’14.

[bib0003] Clark-Price S.C., Lascola K.M., Carter J.E., Da Cunha A.F., Donaldson L.L., Doherty T.J., Hofmeister E.H., Keating S.C.J., Mama K.R., Mason D.E., Posner L.P., Sano H., Seddighi R., Shih A.C., Weil A.B., Schaeffer D.J. (2017). Assessment of agreement among diplomates of the American College of Veterinary Anesthesia and Analgesia for scoring the recovery of horses from anesthesia by use of subjective grading scales and development of a system for evaluation of the recovery of horses from anesthesia by use of accelerometry. Manuel Martin-Flores mv.

[bib0004] Clark-Price S.C., Posner L.P., Gleed R.D. (2008). Recovery of horses from general anesthesia in a darkened or illuminated recovery stall. Veterinary Anaesthesia and Analgesia.

[bib0005] Donaldson L.L., Dunlop G.S., Holland M.S., Burton B.A. (2000). The recovery of horses from inhalant anesthesia: A comparison of halothane and isoflurane. Veterinary Surgery : VS.

[bib0006] Dugdale A.H., Taylor P.M. (2016). Equine anaesthesia-associated mortality: Where are we now?. Veterinary Anaesthesia and Analgesia.

[bib0007] Farmer E., Chase-Topping M., Lawson H., Clutton R.E. (2014). Factors affecting the perception of recovery quality in horses after anaesthesia. Equine Veterinary Journal.

[bib0008] Gerhard T. (2008). Bias: Considerations for research practice. American Journal of Health-System Pharmacy.

[bib0009] Gozalo-Marcilla M., Bettschart-Wolfensberger R., Johnston M., Taylor P.M., Redondo J.I. (2021). Data collection for the fourth multicentre confidential enquiry of perioperative fatalities (CEPEF4) study: New technology and preliminary results. Animals.

[bib0010] Grimes D.A., Schulz K.F. (2002). Bias and causal associations in observational research. Lancet (London, England).

[bib0011] Holman L., Head M.L., Lanfear R., Jennions M.D. (2015). Evidence of experimental bias in the life sciences: Why we need blind data recording. PLOS Biology.

[bib0012] Hubbell J.A.E. (1999). Recovery from anaesthesia in horses. Equine Veterinary Education.

[bib0013] Johnston G., Eastment J., Wood J., Taylor P. (2002). The confidential enquiry into perioperative equine fatalities (CEPEF): Mortality results of phases 1 and 2. Veterinary Anaesthesia and Analgesia.

[bib0014] Laurenza C., Ansart L., Portier K. (2020). Risk factors of anesthesia-related mortality and morbidity in one equine hospital: A retrospective study on 1161 cases undergoing elective or emergency surgeries. Frontiers in Veterinary Science.

[bib0015] Leichsenring F., Abbass A., Hilsenroth M.J., Leweke F., Luyten P., Keefe J.R., Midgley N., Rabung S., Salzer S., Steinert C. (2017). Biases in research: Risk factors for non-replicability in psychotherapy and pharmacotherapy research. Psychological Medicine.

[bib0016] Pollock, N.W. (2020). Managing bias in research. Https : //Doi.Org/10.1016/j.Wem.2020.01.001, 31(1), 1–2. 10.1016/J.WEM.2020.01.001. 32044214

[bib0017] Santiago-Llorente I., López-San Román F.J., Villalba-Orero M. (2021). Demographic, preoperative and anaesthesia-related risk factors for unsatisfactory recovery quality in horses undergoing emergency abdominal surgery. Veterinary Anaesthesia and Analgesia.

[bib0018] Scarabelli S., Rioja E. (2018). Retrospective evaluation of correlation and agreement between two recovery scoring systems in horses. The Veterinary Record.

[bib0019] Suthers J.M., Christley R.M., Clutton R.E. (2011). Quantitative and qualitative comparison of three scoring systems for assessing recovery quality after general anaesthesia in horses. Veterinary Anaesthesia and Analgesia.

[bib0020] Vettorato E., Chase-Topping M.E., Clutton R.E. (2010). A comparison of four systems for scoring recovery quality after general anaesthesia in horses. Equine Veterinary Journal.

[bib0023] Wagner A.E., Mama K.R., Steffey E.P., Hellyer P.W. (2008). A comparison of equine recovery characteristics after isoflurane or isoflurane followed by a xylazine–ketamine infusion. Veterinary Anaesthesia and Analgesia.

[bib0024] Young S.S., Taylor P.M. (1993). Factors influencing the outcome of equine anaesthesia: A review of 1314 cases. Equine Veterinary Journal.

