# Supplementary material for: Influence of the anaesthetist´s perception on the evaluation of recovery quality in horses undergoing general anaesthesia
Source: Vet Anim Sci. 2026 Oct 15;34:100899. doi: 10.1016/j.vas.2026.100899 (PMC13718388; doi:10.1016/j.vas.2026.100899)
Supplement: Supplementary file 1 [file mmc1.docx]

**Figure S1.** Histogram showing the distribution of score differences between video review (VR) and real-time recovery assessment (RT), stratified by time of evaluation: day (8AM-9PM; **A and B**; p<0.0001 for SS; p<0.0001 for DS) and night (9PM-8AM; **C and D**; p<0.05 for SS; non-significant for DS). Differences were calculated as VR-RT. Green bars indicate cases where RT assessment was more optimistic, red bars indicate cases where RT assessment was more pessimistic. Grey bars represent identical scores. For the simple scale (SS; **A, C),** lower scores indicate better recovery quality. For the detailed scale (DS; **B, D),** higher scores indicate better recovery quality.

**Figure S2.** Histogram showing the distribution of score differences between video review (VR) and real-time recovery assessment (RT), stratified by elective (**A and B**; p<0.0001 for SS; p<0.0001 for DS) and emergency (**C and D**; p = 0.001 for SS; p < 0.05 for DS) procedures. Differences were calculated as VR-RT. Green bars indicate cases where RT assessment was more optimistic, red bars indicate cases where RT assessment was more pessimistic. Grey bars represent identical scores. For the simple scale (SS; **A, C),** lower scores indicate better recovery quality. For the detailed scale (DS; **B, D),** higher scores indicate better recovery quality.

**Figure S3.** Histogram showing the distribution of score differences between video review (VR) and real-time recovery assessment (RT), stratified by duration of the procedure: short (≤120 min; **A and B**; p<0.0001 for SS; p<0.001 for DS) and long (>120 min; **C and D**; p<0.0001 for SS; p<0.001 for DS). Differences were calculated as VR-RT. Green bars indicate cases where RT assessment was more optimistic, red bars indicate cases where RT assessment was more pessimistic. Grey bars represent identical scores. For the simple scale (SS; **A, C),** lower scores indicate better recovery quality. For the detailed scale (DS; **B, D),** higher scores indicate better recovery quality.

**Figure S4.** Histogram showing the distribution of score differences between video review (VR) and real-time recovery assessment (RT), stratified by horses with less than 2 attempts to stand (**A and B**; p<0.05 for SS; non-significant for DS) o more than 3 attempts (**C and D**; non-significant for both scales). Differences were calculated as VR-RT. Green bars indicate cases where RT assessment was more optimistic, red bars indicate cases where RT assessment was more pessimistic. Grey bars represent identical scores. For the simple scale (SS; **A, C),** lower scores indicate better recovery quality. For the detailed scale (DS; **B, D),** higher scores indicate better recovery quality.

**Figure S5**. Histogram showing the distribution of score differences between video review (VR) and real-time recovery assessment (RT), stratified by horses that showed overall satisfactory recoveries (**A and B**; p<0.0001 for SS; p<0.001 for DS), regular recoveries (**C and D**; p=0.180 for SS, p<0.05 for DS) and overall unsatisfactory recoveries (**E and F**; non-significant for both scales). Differences were calculated as VR-RT. Green bars indicate cases where RT assessment was more optimistic, red bars indicate cases where RT assessment was more pessimistic. Grey bars represent identical scores. For the simple scale (SS; **A, C, E),** lower scores indicate better recovery quality. For the detailed scale (DS; **B, D, F),** higher scores indicate better recovery quality.
